# Tuberculosis of the parotid gland: histology surprise

**DOI:** 10.11604/pamj.2015.20.343.5673

**Published:** 2015-04-10

**Authors:** Noureddine Errami, Amine Benjelloun, Nessrine Tahtah, Bouchaib Hemmaoui, Ali Jahidi, Ismail Nakkabi, Mohamed Zalagh, Fouad Benariba

**Affiliations:** 1 Ear Nose and Throat unit, Mohammed V Military Hospital, Rabat, Morocco; 2 Pulmonology unit, Avicenne Military Hospital, Marrakech, Morocco; 3 Ear Nose and Throat and maxillofacial surgery unit, Hospital of Specialities, Rabat, Morocco

**Keywords:** Tuberculosis, parotid gland, endemic countries

## Abstract

The Parotid gland is rarely involved in tuberculosis, even in endemic countries. We report a case of a 26 year-old woman with no medical history, who presented with a swelling of the parotid lodge. Pathology performed after surgery found a tuberculous parotitis, and the patient received anti-tuberculous regimen with a satisfactory evolution. We discuss both diagnostic and therapeutic modalities for this infection.

## Introduction

Tuberculosis (TB) is an infectious disease caused by Mycobacterium tuberculosis, which can affect any organ [1]. Even in countries where it is widespread, the parotid gland is rarely involved [2–4]. The diagnosis is not always easy because of the similarities in the presentation with a parotid tumor. Thus, the diagnosis can be a histological surprise as is the present case.

## Patient and observation

**Figure 1:**
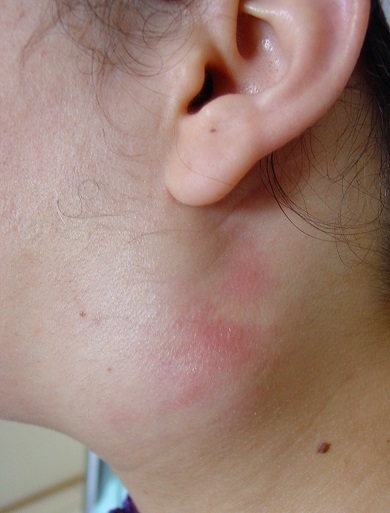
Mass of the left parotid lodge

**Figure 2:**
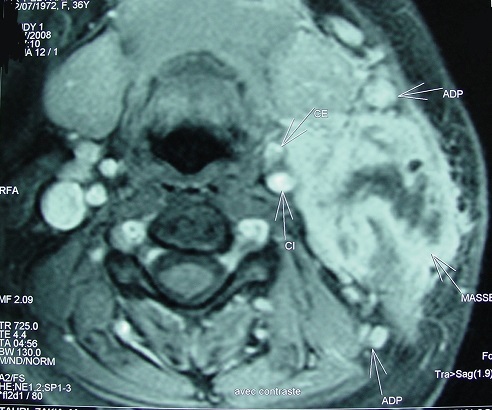
MRI: Heterogeneous mass of the left parotid lodge

**Figure 3:**
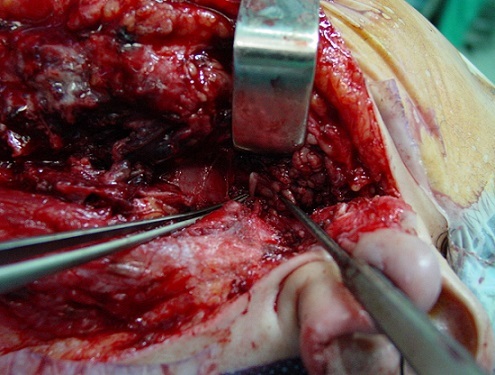
Peroperative image: necrotic parotid tissue

A 26 year-old woman with no medical history of personal or family tuberculosis was admitted for a left sub-mandibular swelling which had appeared 12 months earlier. It began small then gradually increased in volume without alteration of the general condition. Many broad-spectrum antibiotics were attempted without any improvement. Examination showed a 5 cm painless, elastic left parotid mass, adherent to the skin and deeper layers, with mild inflammation of the overlying skin but without fistula or facial paralysis (Figure 1). Moreover, we found ipsilateral sub-mandibular lymphadenopathies. The contralateral parotid region was unremarkable. Otoscopy was normal and oral cavity examination showed neither left tonsillar deviation nor purulent secretions from the left Stensen's duct. The MRI showed a 4.6 cm, irregular and heterogeneous tumoral process of the deep lobe of the left parotid gland with a T1 hyposignal and a T2 hypersignal, and heterogeneous enhancement after gadolinium injection (Figure 2). Fine needle aspiration cytology (FNAC) of the mass was inconclusive. We, therefore, decided an exploratory extrafacial parotidectomy. In preoperative, parotid tissue appeared necrotic and purulent by places (Figure 3). Frozen section showed tuberculous lesions in both parotid and sub-mandibular lymph nodes. We, therefore, restricted surgery to a left lower pole parotidectomy. A check-up looking for second location tuberculosis (clinical examination, chest X ray, sputum analysis) was negative. The tuberculin skin test was positive at 12 mm. A prolonged (9 months) four drug antituberculous regimen (H: isoniazid, E: ethambutol, R: rifampicin and Z: pyrazinamid) was then started according to our usual protocol: 2 RHZE/7 RH. The evolution was satisfactory with no relapse after six years.

## Discussion

TB is a necrotizing granulomatous disease affecting mostly the lungs. Extrathoracic locations of the disease represent 20 percent of all cases. Lymph node tuberculosis is the most common extrathoracic form [3, 5, 6]. Tuberculosis of the parotid gland remains extremely rare, even in countries where TB is endemic. The salivary glands are usually spared of tuberculosis because of thiocyanate ions and proteolytic enzymes such as lysozyme that confer antibacterial action. In addition, the continuous flow of saliva prevents stagnation and growth of mycobacteria (important inhibitory factor). Infection of parotid tissue can be by direct passage of bacillus from the oral cavity via the drainage channel of the gland. Haematogenous or lymphatic spread of the infection is also possible [2]. 25 percent of patients with parotid tuberculosis have a concomitant pulmonary infection [4]. The clinical presentation is variable, when lymph nodes of the parotid lodge are involved; it usually appears as a localized parotid mass gradually increasing in size over months or years [7–9]. It may also present as a parotitis with diffuse swelling of the gland. In the latter form, invasion involves glandular parenchyma. Parotid abscesses or peri-auricular fistula have also been described [10]. In chronic swelling, unilateral tuberculous parotitis is clinically indistinguishable from a benign or malignant tumour and the diagnosis is histological [1]; especially in the absence of pulmonary infection [9]. The differential diagnosis should also include: actinomycosis, suppurative parotitis, mumps, sarcoidosis and Sjogren′s syndrome [1, 11]. The most useful examinations for the study of the parotid parenchyma are ultrasound, computed tomography and magnetic resonance imaging (MRI). They can identify malignancy signs and distinguish intraparotid from extraparotid locations [2]. In parotid MRI, TB appears as a T1 hyposignal and T2 hypersignal [12]. The definitive diagnosis of tuberculosis depends on isolation and identification of mycobacteria from a collection of parotid tissue [5, 7]. The discovery of PCR has allowed clinicians to make a quick diagnosis, in less than 12 hours [1]. FNAC is recommended as a useful and reliable technique for the diagnosis of the parotid gland tuberculosis [3, 5]. It has a high sensitivity and specificity in the diagnosis of TB [8]. Handa reported five cases of parotid TB documented by FNAC and medically treated [13]. However, FNAC has only value when positive. Exploratory parotidectomy becomes essential when cytology is non-contributory [5]. In a meta-analysis by Lee and Liu on 49 cases of parotid TB, diagnosis was established by parotidectomy in 34 cases (69%) [3, 5]. When the diagnosis is obtained by minimally invasive methods, surgery becomes unnecessary and anti-tuberculous chemotherapy is sufficient to obtain resolution. The medical treatment consists of the combination of powerful anti-tuberculous drugs according to the regimen: 2RHZ/4RH for a period of 6 to 9 months.

## Conclusion

Tuberculosis of the parotid gland is rare, with atypical clinical presentations. It must always be kept in mind as a differential diagnosis in any parotid swelling especially in endemic countries.
